# Augmented COlorimetric NANoplasmonic (CONAN) Method for Grading Purity and Determine Concentration of EV Microliter Volume Solutions

**DOI:** 10.3389/fbioe.2019.00452

**Published:** 2020-02-12

**Authors:** Andrea Zendrini, Lucia Paolini, Sara Busatto, Annalisa Radeghieri, Miriam Romano, Marca H. M. Wauben, Martijn J. C. van Herwijnen, Peter Nejsum, Anne Borup, Andrea Ridolfi, Costanza Montis, Paolo Bergese

**Affiliations:** ^1^Department of Animal Science, Food and Nutrition-Università Cattolica del Sacro Cuore, Piacenza, Italy; ^2^Consorzio Interuniversitario Nazionale per la Scienza e la Tecnologia dei Materiali, Florence, Italy; ^3^Department of Molecular and Translational Medicine, University of Brescia, Brescia, Italy; ^4^Consorzio Sistemi a Grande Interfase, Department of Chemistry, University of Florence, Sesto Fiorentino, Italy; ^5^Department of Transplantation, Mayo Clinic, Jacksonville, FL, United States; ^6^Department of Biochemistry and Cell Biology, Faculty of Veterinary Medicine, Utrecht University, Utrecht, Netherlands; ^7^Department of Clinical Medicine, Faculty of Health, Aarhus University, Aarhus, Denmark; ^8^Istituto per lo Studio dei Materiali Nanostrutturati (CNR-ISMN), Bologna, Italy; ^9^Department of Chemistry, University of Florence, Sesto Fiorentino, Italy

**Keywords:** extracellular vesicles, synthetic vesicles, liposomes, purity, titration, particle number, nanoparticles, nanoplasmonics

## Abstract

This protocol paper describes how to assign a purity grade and to subsequently titrate extracellular vesicle (EV) solutions of a few microliters in volume by microplate COlorimetric NANoplasmonic (CONAN) assay. The CONAN assay consists of a solution of gold nanoparticles (AuNPs) into which the EV preparation is added. The solution turns blue if the EV preparation is pure, whereas it stays red if soluble exogenous single and aggregated proteins (SAPs; often referred to as protein contaminants) are present. The color change is visible by the naked eye or can be quantified by UV-Vis spectroscopy, providing an index of purity (a unique peculiarity to date). The assay specifically targets SAPs, and not the EV-related proteins, with a detection limit <50 ng/μl (an order of magnitude higher resolution than that of the Bradford protein assay). For pure solutions, the assay also allows for determining the EV number, as the color shift is linearly dependent on the AuNP/EV molar ratio. Instead, it automatically reports if the solution bears SAP contaminants, thus avoiding counting artifacts. The CONAN assay proves to be robust and reliable and displays very interesting performances in terms of cost (inexpensive reagents, run by standard microplate readers), working volumes (1–2 μl of sample required), and time (full procedure takes <1 h). The assay is applicable to all classes of natural and artificial lipid microvesicles and nanovesicles.

## Introduction

Cell communication is branched and complex. Over the past years, the conveyance of information by several “nanoscale” routes has been shown to be a key mechanism in numerous biological processes (Baker, 2017; Maas et al., 2017). Among others, the secretion of membranous bio-nanoparticles called extracellular vesicles (EVs) has gained increasing attention. Such soft particles are composed of a lipid bilayer trimmed for safe and specific long-range transport and a hydrophilic core in which bio-macromolecules are stored. EVs exhibit biomedical properties superior to even the most innovative synthetic nanomaterials, providing an array of possible applications in medical treatment and diagnostics, including regenerative medicine, immunology, neuroscience, microbiology, bio-nanotechnology, pharmacology, and others (De Toro et al., 2015; Stremersch et al., 2016; Vader et al., 2016; Paolini et al., 2018).

However, EV biology investigation and clinical translation are not adequately supported by current manufacturing and characterization technologies (Margolis and Sadovsky, 2019; Wiklander et al., 2019). In particular, attribution of a purity grade and determination of the molar concentration (particle number) of EV preparations in a reproducible and scalable/cost-effective fashion requires further improvement (Thery et al., 2018).

Purity with respect to soluble exogenous single and aggregated proteins (SAPs, often referred to as protein contaminants) is to date expressed through ratios of components that may be associated or not to EVs and obtained through different quantification methodologies, for example, protein/particle ratio or protein/lipid ratio. The applicability of these methods still needs to be fully established and, in any case, they do not allow to assign an absolute grade to the purity of the EV preparation, which is univocally related to the exogenous protein content (Thery et al., 2018).

Investigation of particle number greatly employs light scattering techniques. Among the technologies available, the most diffused are nanoparticle tracking analysis (NTA) (McNicholas and Michael, 2017), flow cytometry (Tian et al., 2018), and dynamic light scattering (DLS) (Montis et al., 2017). However, particle quantification by light scattering is often biased by poor distinction between EV and other co-isolated bio-nanoparticles (protein aggregates, lipoproteins), sample polydispersity, or intrinsic low sensitivity due to particle size.

Other techniques, such as fluorescence correlation spectroscopy (FCS) (Montis et al., 2017), resistive pulse sensing (RPS) (de Vrij et al., 2013), or cryo-electron microscopy (Arraud et al., 2014), allow to circumvent some of the hurdles imposed by the methods described above but are nevertheless affected by other drawbacks, including instrumental limitations due to pore size in RPS or artifacts caused by fluorophore micelles and/or particles in FCS.

In this paper, we present a step-by-step protocol to assign a purity grade and to subsequently titrate EV formulations by an augmented microplate version of the COlorimetric NANoplasmonic (CONAN) assay (Maiolo et al., 2015; Mallardi et al., 2018; Picciolini et al., 2018; Gualerzi et al., 2019; Rojalin et al., 2019) with an optimized balance between robustness and accessibility.

The advantages and limitations with respect to the current methods and instruments will be highlighted in a dedicated section (Section Advantages and Limitations). We anticipate that very good sensitivity to SAPs, use of exceptionally cost-effective reagents and a standard microplate reader, few microliters of working volumes, and <1 h analysis time make the CONAN assay highly appealing. In addition, it is in principle applicable/extendable to lipid microvesicles and nanovesicles other than EVs, including other membranous biogenic particles, drug loaded-targeted liposomes, and artificial EVs.

The assay consists of a nanomolar red-colored solution of gold nanoparticles (AuNPs) mixed with an EV preparation, and its principle relies on the AuNP high surface energy. Specifically, AuNPs mixed with SAPs are preferentially cloaked and passivated by the EVs+SAPs in solution, whereas AuNPs mixed with pure EVs cluster onto their membrane. Because EV separation is a theoretical principle difficult to achieve due to the complexity of biological fluids, all separation and concentration methods result in heterogeneous formulations containing EVs and SAPs at different ratios and therefore suitable to be tested by the CONAN assay. AuNPs clustered onto the EV surface shift and broaden their localized surface plasmon resonance (LSPR) peak, resulting in a visible change in the color of the solution from red to blue. Contrarily, AuNP surface passivation from SAPs prevents their aggregation and any resulting LSPR shift. Conclusively, the change of the color of the solution is directly related to the grade of purity of the EV formulation, and it can be assessed by the naked eye and/or quantified by UV-Vis spectroscopy [the aggregation index (AI)]. The CONAN assay also serves for the titration of the total molar concentration of high-purity grade EV preparations, as in this case, the AI is linearly dependent on the AuNP/EV molar ratio. This next assay needs building of a calibration curve with reference liposome solutions.

## Materials and Equipment

A complete list of reagents and equipment required for the CONAN assay and preparatory steps is reported in Tables 1, 2, respectively.

**Table 1:** Reagents.

**Name of reagent**	**Manufacturer**	**City and country**
1-palmitoyl-2-oleoyl-sn-glycero-3-phosphocholine (POPC)	Avanti Polar Lipids	Alabaster, AL, USA
Chloroform 100%	Sigma-Aldrich	St. Louis, MO, USA
Methanol 100%	Sigma-Aldrich	St. Louis, MO, USA
Sterile PBS	Lonza	Basel, Switzerland
Trisodium citrate · 2H_2_O (≥99%)	Sigma-Aldrich	St. Louis, MO, USA
HAuCl_4_ · 3H_2_O	Sigma-Aldrich	St. Louis, MO, USA
HPLC grade water	Sigma-Aldrich	St. Louis, MO, USA

**Table 2:** Equipment.

**Name of reagent**	**Manufacturer**	**City and country**
Sonopuls HD 2070 sonicator	Bandelin Electronic GmbH & Co.	Berlin, Germany
5417 C centrifuge	Eppendorf	Hamburg, Germany
MR 300IK stirrer/heatplate	Heidolph	Schwabach, Germany
EnSight multimode reader^*^	Perkin-Elmer	Waltham, MA, USA
BI 9000 AT DLS	Brookhaven instruments corporation	Holtsville, NY, USA

* *No specific reader is needed. A standard microplate UV-Vis reader that is able to collect absorbance from 450 to 900 nm is sufficient*.

## Methods

The CONAN preparatory procedures consist of the synthesis of AuNPs and liposomes used in the assay. Such colloidal solutions are stable for up to 1 month if properly synthesized and stored, thus it is not necessary to process new batches every time the CONAN assay is performed. However, periodic control of AuNPs and liposomes is advised to monitor their respective stability over time. Preparatory procedures are here presented stepwise. Use of commercial liposomes and AuNPs has never been investigated but could represent a functional, time-saving option.

### Preparatory Procedures

#### Palmitoyl-2-Oleoyl-Sn-Glycero-3-Phosphocholine (POPC) Liposome Preparation

In the CONAN assay, POPC liposomes are used as synthetic, pure, and convenient mimics of EVs to create the calibration line used for EV titration.

- Dissolve the POPC in a glass vial in a chloroform-methanol 6:1 solution to a final concentration of 10 mM. **Crucial step:** Vial diameter/lipid concentration ratio is important to ensure a smooth and homogeneous lipid film; for a final reaction volume of 1 ml (7.5 mg of POPC dissolved in 6:1 chloroform-methanol), the use of a round-bottom, 20-mm ø glass vial is advised.
- Evaporate the organic solvent in a fume hood under a dry stream of nitrogen or compressed air using a glass Pasteur pipette until a thin lipid film can be observed on the vial's inner surface. **Crucial step:** To obtain a lumpless lipid film, it is recommended to continuously slowly rotate the vial during solvent evaporation. Also, hold the tip of the pipette at least at 3–4 cm from the vial's bottom.
- Dry the vial overnight under vacuum to remove all of the solvent.
- Add sterile, warm PBS (1×; 50°C) to a final lipid molar concentration of 10 mM.
- Vortex the vial for 2 min to hydrate the lipid film until a white solution of multilamellar vesicles is obtained. Incubate the vial for 2 min at room temperature. Repeat this step two more times.
- Transfer the solution to a clean plastic tube and tip-sonicate it for 30 min at 10 W. **Crucial step:** Tip-sonication produces heat; cool down the solution with ice during this procedure to prevent lipid degradation and limit PBS evaporation.
- Transfer the solution to an Eppendorf tube and centrifuge at 800 *g* for 10 min. **Crucial step:** Centrifugation is necessary to eliminate microscopic sonication debris. Note that 800 *g* will not pellet phospholipids and/or liposomes, which therefore remain in the supernatant.
- Collect the supernatant and store it at 4°C in a plastic tube. Liposome solutions can be stored for up to 1 month without significant change in liposome size distribution. **Crucial step:** Extended tip-sonication often leads to solvent evaporation; add fresh sterile PBS to compensate for the loss.
- Check liposome size and size distribution. The use of an optical technique, such as dynamic light scattering (DLS), nanoparticle tracking analysis (NTA), or atomic force microscopy (AFM), is advised. Usually, this procedure results in liposomes of 85–115 nm ø, with a polydispersity index (PDI) ≤0.10.

Note: POPC is here employed as a model lipid, but its use is not exclusive. Keep in mind that some of the conditions reported could need some tuning (e.g., different solvent mixture) if another phospholipid, or a mixture of lipids, is used for liposome synthesis.

Also notice that liposomes can be produced through extrusion instead of sonication with similar results.

#### AuNP Synthesis

AuNPs are synthesized through classic Turkevich's citrate reduction method (Turkevich et al., 1951).

- Dissolve trisodium citrate · 2H_2_O in HPLC grade water to a final concentration of 34.0 mM (1.0% wt).
- Prepare 20 ml of 1 mM HAuCl_4_ in HPLC grade water.
- Boil the HAuCl4 solution under continuous stirring.
- Inject 2 ml of trisodiumcitrate · 2H_2_O 34.0 mM into the boiling solution. **Crucial step**: trisodium citrate injection speed influences AuNP size and therefore final concentration. A one-shot injection leads to smaller particles (usually around 12-15 nm); slow injection will result in NP aggregation and precipitation.
- Keep stirring and wait for the solution to change color from the original pale yellow to wine red.
- After 10 min, cool the solution in a water-ice bath for 5min.
- Store the AuNPs at 4°C. **Crucial step**: 10–20 nM AuNP solutions are metastable and tend to form aggregates and precipitate with time. In our experience they keep their properties with respect to the assay up to 1 month of storage at the above described conditions. Anyhow it is strongly suggested to freshly (re)determine before any usage the key characteristics of the AuNP solution which are relevant for the assay as described in section Determination of AuNP Molar Concentration and Spectral Properties.

### Determination of AuNP Molar Concentration and Spectral Properties

AuNP molar concentration can be determined in many fashions. DLS is the most diffused technique. UV-Vis spectroscopy is also a viable alternative, with the advantage to be simpler and more affordable than DLS. UV-Vis spectroscopy also provides insights on AuNP spectral properties at a glance. In the following, we will refer to Haiss et al. (2007) for the protocols for determination of the AuNP size and concentration by UV-Vis spectroscopy (and we redirect the interested reader for theoretical and experimental details). In Section (Anticipated) Results and Discussion (**Figures 2A,B**), results from both DLS and UV-Vis characterization will be presented.

### CONAN Assay

#### Calibration Line

**Figure 1:**
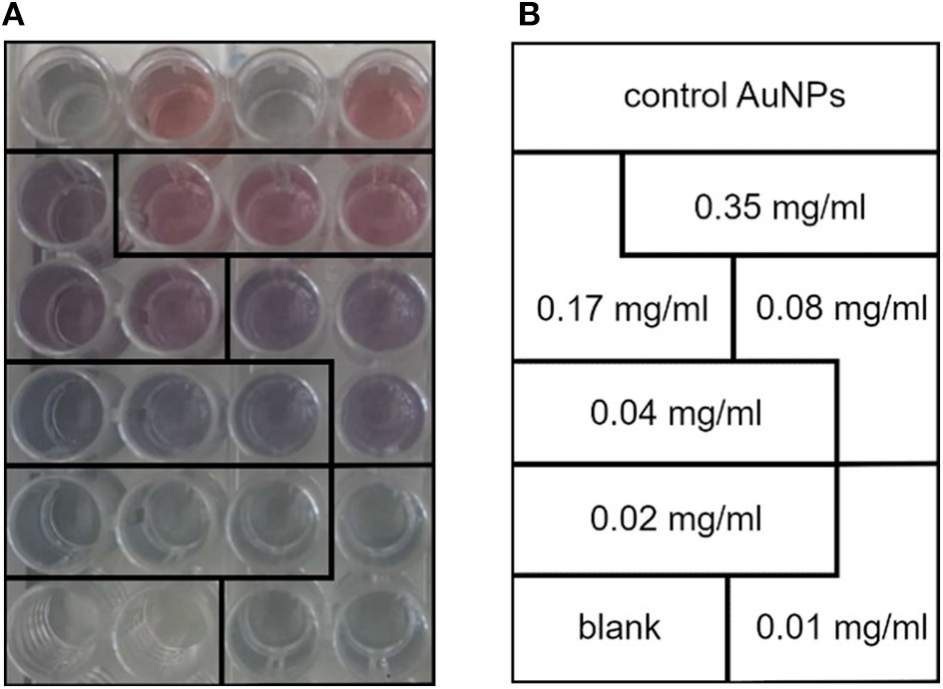
**(A)** Calibration line standards made with different concentrations of 1-palmitoyl-2-oleoyl-sn-glycero-3-phosphocholine (POPC) liposomes. Notice how the gold nanoparticle (AuNP) color gradually turns from red to blue with the decrease in POPC concentration. **(B)** Lipid concentration of the standards used to plot the COlorimetric NANoplasmonic (CONAN) calibration line. The scheme retraces the sample disposition shown in **(A)**.

**Figure 2:**
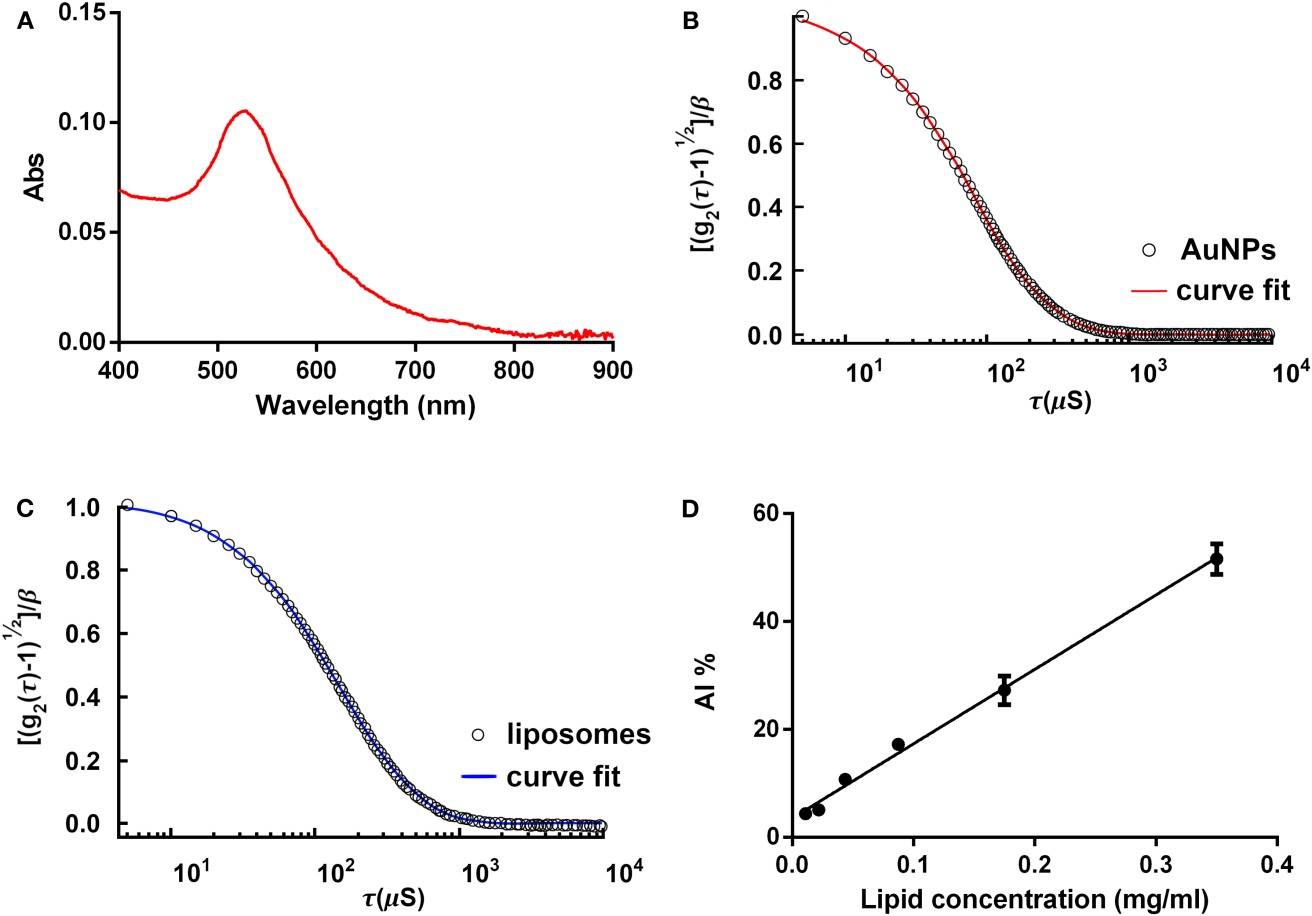
COlorimetric NANoplasmonic (CONAN) assay, preparatory procedures. **(A)** Gold nanoparticle (AuNP) UV-Vis spectrum. The single, sharp absorption peak indicates that the AuNPs are monodispersed. **(B)** The dynamic light scattering (DLS) autocorrelation function of the AuNPs. Function decay and curve shape suggest the presence of monodispersed AuNPs, sized ~15 nm. **(C)** The DLS autocorrelation function of POPC liposomes. Data extrapolated from the curve indicate that liposomes are 100 ± 15 nm. **(D)** The CONAN calibration line obtained plotting the AI ratio of liposome standards with the related lipid concentration (*r*^2^ = 0.98).

1. Prepare standards at decreasing concentration by diluting the liposome stock solution previously prepared and characterized. **Crucial step:** A proper calibration line needs the preparation of at least five standards with decreasing phospholipid concentration, and each measurement should be performed in triplicate. Calibration conditions have been optimized using standards ranging from 0.35 to 0.01 mg/ml phospholipids. Changes in quantification range can be done by adjusting nanoparticles vs. POPC concentration. Mind that a new calibration line should be created each time a new batch of AuNPs is synthesized.
2. Add 25 μl of each standard to a 96-multiwell plate.
3. Add 50 μl of AuNP 6 nM to each well.
4. Add 25 μl of sterile filtered PBS (1×) to each well and mix gently. A gradual change (from red to blue, related to POPC concentration, Figures 1A,B) of solution color should be noticed.
5. Incubate the plate at room temperature for 30 min.
6. Put the plate in a microplate reader, and collect the absorbance spectrum from 400 to 900 nm.
7. Extrapolate AuNP AIs from the spectra of standards. AI is a useful tool to calculate NP aggregation state. For spherical gold NPs, AI is described as the ratio of the absorbance at LSPR peak of pure, monodispersed NPs and the absorbance at some significant red-shift wavelength
  (1) $$\begin{array}{l} AI=\;\frac{Abs_{LSPR}}{Abs_{650nm}+Abs_{850nm}} \end{array}$$
  The LSPR wavelength depends on the AuNP size and can be easily monitored with UV-Vis spectroscopy, whereas red-shift wavelength is here described as the absorbance at 650 nm plus the absorbance at 850 nm. Whenever the spectrophotometer used for spectra collection could not measure the absorbance at 850 nm, it is possible to substitute such value with the absorbance measured at 800 or 780 nm, with minimal discordance in terms of EV quantification (<1%; see a comparison between different calibration lines in Figure S1).
  It is also possible to express the AI of samples as a percentage of the ratio between the AI of the sample and the AI of pure, monodispersed AuNPs:
  (2) $$\begin{array}{l} AI\;ratio=\frac{AI_{Sample}}{AI_{AuNPs}}\;\% \end{array}$$
8. Plot each standard's AI ratio vs. the related phospholipid concentration, and create a calibration line (Figure 2D) (Mallardi et al., 2018).

#### Reference Points

Three reference samples and points are required. Careful preparation and use, and correct understanding of the reference points are *conditio sine qua non* for proper functioning of the assay:

- The blank water sample. It consists of 100 μl of HPLC grade water. Its absorbance spectrum needs to be measured and subtracted from the spectra of all of the samples, the next controls included (as in any UV-Vis spectroscopy standard procedure).
- The reference monodispersed AuNP sample (normREF). This is to be prepared by gently mixing 50 μl of AuNPs 6 nM + 50 μl of HPLC grade water. The AI of this sample (AI_AuNPs_) sets the maximum AI for the particular assay in use and is used to normalize the EV AIs that is used to calculate the AI ratios [Equation 2].
- The “no EV” sample (intREF). This is to be prepared by gently mixing 25 μl of HPLC grade water + 50 μl of AuNPs 6 nM and 25 μl of PBS. The AI ratio of the intREF defines the threshold below which the spectral red shift (viz. the color change into blue of the solution) is only due to interaction between the AuNPs and the EVs. Its value should be 45–50%. Note: The AI ratio of the pure samples must be lower, or get lower along with dilution, than the AI ratio of the intREF.

#### EV Purity Check

1. Put 23 μl of HPLC grade water + 2 μl of sample in a 96-multiwell plate. **Crucial step:** To our experience, most EV samples are too concentrated to be correctly assessed with the CONAN assay and therefore must be diluted. Dilution is a key factor for the assay to work and should be performed with HPLC grade water, not with PBS. The right dilution must be empirically evaluated, considering sample features.
2. Add 50 μl of AuNP 6 nM.
3. Add 25 μl of PBS (1×), and mix gently.
4. Repeat points 1 → 3 in triplicate for each sample.
5. Incubate the plate at room temperature for 30 min.
6. Put the plate in a microplate reader, and collect the absorbance spectrum from 400 to 900 nm.
7. Extrapolate sample AIs from the spectra using Equation (1), and calculate AI ratio using Equation (2). Samples with an AI ratio ≤20% contain low levels of soluble contaminants [below 0.05 μg/μl, which is the SAP limit of detection (LOD) for the CONAN assay] and are therefore considered highly pure. SAP LOD calculation experiment is explained and shown in Figure S2.
8. Interpolate the AI ratio with the calibration line to calculate the content of lipids in the tested EV sample. The content of lipids is directly linked to the EV surface area and is necessary for the plasmonic titration of the EV preparations. **Crucial step:** A reliable titration can be performed only on pure samples, namely, the ones whose AI ratio is ≤20%.

#### Estimation of EV Concentration

The EV concentration calculation through the proposed model is based on several assumptions that will be further discussed in Section Advantages and Limitations.

Two parameters must be defined prior to the EV titration:

- EV mean diameter obtained through DLS, NTA, or AFM
- The number of lipids per vesicle (*N*_l_) was calculated using the equation (Friedrich et al., 2017):

(3) $$\begin{array}{l} N_{l}=\frac{2\pi d^{2}}{a} \end{array}$$

where:

*d* = EV mean diameter [measured with DLS, NTA, or atomic force microscopy (AFM)].
*a* = mean phospholipid's head cross section, which can be assumed to be equal to 0.65 nm^2^ (Lantzsch et al., 1994).
The EV molar concentration can be now estimated stoichiometrically using Equation (4).

(4) $$\begin{array}{l} EVs_{\left[M\right]}=\frac{l_{CONAN}}{POPC_{M}N_{l}} \end{array}$$

where:

*EVs*_[*M*]_ = EVs molarity
*l*_*CONAN*_ = lipid concentration in your sample calculated with the CONAN assay
*POPC*_*M*_ = POPC molar mass
*N*_*l*_ = number of lipids per vesicle obtained from Equation (3)
The procedure to obtain Equation (4) is reported step-by-step in the following.

First, calculate the molarity of POPC in the solution:

(5) $$\begin{array}{l} POPC_{\left[M\right]}=\frac{l_{CONAN}}{POPC_{M}} \end{array}$$

where:

*l*_*CONAN*_ = lipid concentration in your sample calculated with the CONAN assay
*POPC*_*M*_ = POPC molar mass

Next, convert POPC_[M]_ into molecules of POPC per liter of solution:

(6) $$\begin{array}{l} \frac{POPC}{L}=POPC_{\left[M\right]}N_{a} \end{array}$$

where:

*N*_*a*_ = Avogadro's constant
*POPC[M]* = POPC molarity

Third step, calculate the EVs per liter of solution:

(7) $$\begin{array}{l} \frac{EVs}{L}=\frac{\frac{POPC}{L}}{N_{l}} \end{array}$$

where:

*N*_*l*_ = Number of lipids per vesicle obtained‘ from Equation (3)
*POPC/L* = POPC molecules per liter of solution

Note: Equation (7) can also be intuitively used to calculate the EV number in any given volume.

Now, convert EVs/L into EV molarity:

(8) $$\begin{array}{l} EVs_{\left[M\right]}=\frac{\frac{EVs}{L}}{N_{a}} \end{array}$$

where:

*EVs/L* = EVs per liter of solution
*N*_*a*_ = Avogadro's constant

Finally, Equation (4) can be extracted from Equation (8) through a simple substitution:

$$\begin{array}{l} EVs_{\left[M\right]}=\frac{\frac{EVs}{L}}{N_{a}}=\frac{\frac{POPC}{L}}{N_{l}N_{a}}=\frac{POPC_{\left[M\right]}N_{a}}{N_{l}N_{a}}=\frac{l_{CONAN}}{POPC_{M}N_{l}} \end{array}$$

## (Anticipated) Results and Discussion

A working example of the CONAN assay preparatory procedures, together with its application to EV samples of relevant interest, is given in this section. The assayed EVs were separated from bovine milk and *Ascaris suum* (*A. suum*) culture medium.

Milk EVs were separated by following two procedures: there are the ones hereafter referred to as milk-contaminated EVs by iodixanol gradient centrifugation and size exclusion chromatography and those hereafter referred to as milk-pure EVs by starting with acidification, then followed by iodixanol gradient centrifugation and size exclusion chromatography. *A. suum* EVs (hereafter referred to as *A. suum* pure EVs) were obtained by concentrating the culture medium with Amicon® centrifugal filters followed by size exclusion chromatography. EVs were characterized according to MISEV 2018 guidelines (Thery et al., 2018). Relevant to this protocol paper, the EV average size was estimated through in-liquid AFM measurements (**Figure 4**). Full details on the EV separation protocols and characterization are given in Ridolfi et al. (2019).

### Preparatory Procedures

Figure 2A shows the characterization of freshly synthesized, citrate-capped AuNPs, performed with the UV-Vis spectroscopy. LSPR absorption peak is centered at 519 nm. The AuNP size and concentration were estimated to be ~14 and 18.2 nM, respectively, according to Haiss et al. (2007). No secondary peaks are present in the AuNP absorption spectrum, thus excluding NP subpopulations with different sizes or shapes other than spherical.

In Figure 2B, DLS characterization of the very same AuNPs is shown. Results are in accordance with UV-Vis spectroscopy. The autocorrelation function suggests the presence of a single population of AuNPs of ~15 nm in size.

Figure 2C reports DLS characterization of reference liposomes. Autocorrelation function indicates that liposomes measure 100 ± 15 nm, as expected following the protocol described in Section Palmitoyl-2-Oleoyl-Sn-Glycero-3-Phosphocholine (POPC) Liposome Preparation.

Figure 2D reports the calibration line obtained by linearly fitting the AI ratio of liposome standards vs. lipid concentration. Using the described experimental conditions (POPC liposomes probed with 6 nM AuNPs), the relation between AI ratio and lipid concentration was linear in the range of 0.01–0.35 mg lipids/ml (*r*^2^ = 0.98).

### CONAN Assay

**Figure 3:**
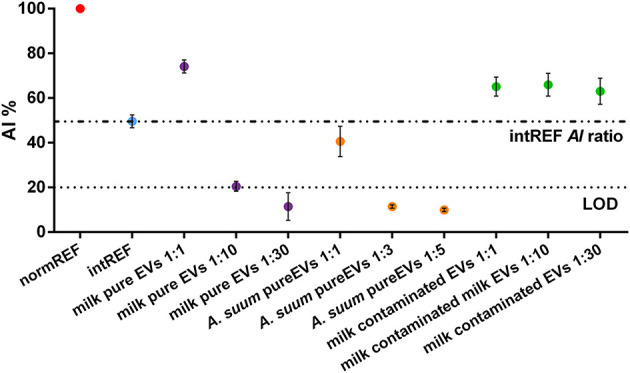
Aggregation index (AI) ratios of pure and contaminated extracellular vesicle (EV) samples. The intREF AI ratio defines the threshold below which the spectral red shift is only due to the interaction between the AuNPs and the EVs (dashed-dotted line). The dotted line represents the CONAN assay threshold for SAP detection (<20% AI ratio means that the soluble protein content is ≤0.05 μg/μl). Further information about the LOD calculation is found in the Supporting Materials.

Figure 3 shows the AI ratio of different EV formulations. Each EV sample was measured in triplicate. The normREF (monodispersed AuNPs) AI ratio (red dot) is used as AI ratio reference value (100%). The intREF sample (“no EV” sample, cyan dot) defines the threshold below which it is only due to the interaction between the AuNPs and the EVs: If the tested sample's AI ratio is similar to the intREF AI ratio and does not decrease upon sample dilution, your preparation does not contain a sufficient number of EVs to trigger AuNP clustering.

When measuring EV purity with the CONAN assay, three different cases can occur:

**- CASE 1:** The sample is pure but very concentrated. The AI ratio of such a sample is above the intREF AI ratio (evidenced by the dashed-dotted line). However, it decreases below it upon dilution. This case is well-represented by the milk-pure EV sample (purple dots). In the pure, undiluted sample (first purple dot), the AI ratio is much higher than 20%, which is the CONAN assay LOD (Section Microplate UV-Vis Spectroscopy), represented by the dotted line. Upon dilution, the AI ratio readily falls below 20% (third purple dot), pointing out that the sample is clean from any detectable SAP. Plasmonic titration can be performed.
**- CASE 2:** The sample is pure. The AI ratio is between the intREF AI ratio and the LOD. This case is represented by *A. suum* pure EV sample (orange dots). The AI ratio of diluted samples behaves exactly as for **CASE 1**, but a smaller dilution factor is needed to drop below LOD, meaning that *A. suum* EVs are less concentrated than milk EVs. Plasmonic titration can be performed.
**- CASE 3:** The sample is contaminated by SAPs. The AI ratio of such a sample is above the intREF AI ratio (dashed-dotted line) and keeps constant upon dilution. In a contaminated formulation of EVs, the AI ratio usually stabilizes around 60–70% regardless of the dilution factor used to test it because the sample contains a high amount of SAPs that dominate the interaction with the AuNPs, coating them, and inhibiting clustering at the EV membrane. This case is represented by the milk-contaminated EV sample (green dots). Plasmonic titration cannot be performed.

This preparation was obtained by a coarser separation with respect to the protocol used to obtain the CASE 1 sample (milk-pure EV sample); hence, presence of SAPs was expected.

**Figure 4:**
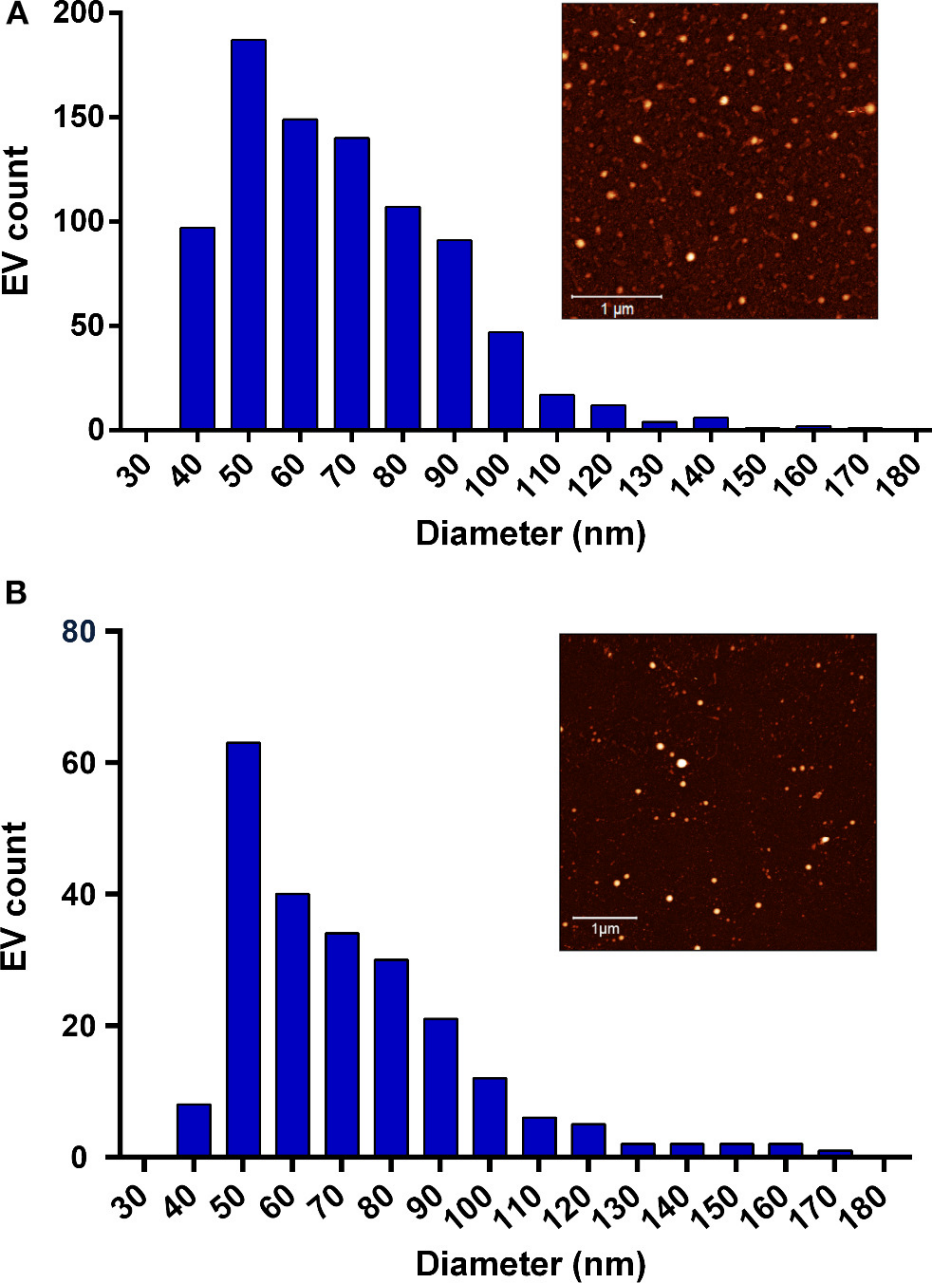
Size distribution of the tested extracellular vesicles (EVs). **(A)** The Milk-pure EV size distribution extrapolated from the in-liquid atomic force microscopy (AFM) analysis. EV mean size = 68.2 nm (measurements performed on 861 EVs). The sample AFM image is shown in the inset (scale bar 1 μm). **(B)** The *A. suum* pure EV size distribution extrapolated from in-liquid AFM analysis. EV mean size = 71.5 nm (measurements performed on 229 EVs). The sample AFM image is shown in the inset (scale bar 1 μm).

In Figures 4A,B, milk-pure EV and *A. suum* pure EV size distributions are presented. The EV mean size (68.2 nm for milk EVs; 71.5 nm for *A. suum* EVs) was measured using AFM, analyzing at least 10 AFM pictures of EVs adsorbed onto glass substrates coated with poly-L-lysine. Eight hundred sixty-one and 229 EVs were analyzed for milk and *A. suum*, respectively. The mean size is required to estimate EV concentration in pure samples using Equation (3). According to plasmonic titration, the pure EVs from milk sample have a lipid concentration of 15.4 mg/ml, equal to 3.14 · 10^14^ particles/ml (5.21 · 10^−7^ M), whereas the *A. suum* EVs have a lipid concentration of 1.13 mg/ml, equal to 2.07 · 10^13^ particles/ml (3.45 · 10^−8^ M). Due to a high level of proteins in the solution, it was not possible to quantify the SAP-contaminated milk sample using the CONAN assay.

### Troubleshooting Guide

In this section, the most common troubles and frequently asked questions (FAQs) related to the CONAN assay are given and addressed one by one with possible solutions.

#### AuNP Synthesis Fails. Particles Aggregate and Precipitate During Synthesis

In AuNP synthesis, water is usually the culprit. Deviations in pH (from 7) or the presence of salt (electrolytes) in solution can lead to failure. Verify the water source and/or change it.

#### Can the CONAN Assay Work With a Different Type of AuNP?

The CONAN assay has been designed and optimized using 15-nm spherical AuNPs capped with citrate. The use of different AuNPs (e.g., with different size, shape, or surface charge) has never been tested.

#### The AI Ratio of the Liposome Standard Is Too Low (Namely, the Most Concentrated Standard Has an AI Ratio ~20%)

Synthesize a new batch of liposomes, and plot a new calibration line. From our experience, the AI ratio of the most concentrated standard (0.35 mg of lipids/ml) should be attested to ~50%.

#### Can the CONAN Results Be Compared Using Different Batches of AuNPs?

The CONAN results obtained using different batches of AuNPs shall be consistent provided the used AuNPs have consistent characteristics, that is, exhibit overlapping spectral features.

#### How Do I Discriminate AuNP Aggregation Caused by PBS From That Caused by EV Membrane?

This problem is easily addressed by setting up a proper control during EV purity check: the intREF (no EV sample, refer to Section Microplate UV-Vis Spectroscopy, paragraph Reference points). AI ratio of pure tested samples should drop below the intREF AI ratio and the LOD upon dilution.

#### Why Can't I Detect Any Significant Change in Assay Color?

Two main reasons

1. Pure but very concentrated samples result in AI ratios >20%. To minimize misleading conclusions, test different dilutions of the same EV sample and check for noteworthy changes in the AI ratio. Indeed, in pure EV samples, the AI ratio should decrease under the LOD (Figure 3, purple and orange dots). As stated in Section Microplate UV-Vis Spectroscopy, the dilution factor must be empirically evaluated, considering sample features. The advised sample dilutions are 1:10, 1:30, and 1:100.
2. The sample is contaminated by SAPs. In such a case, the AI ratio remains almost constant even when the sample is highly diluted. Try to optimize your EV separation protocol. Note that a contaminated sample normally has a very high AI ratio (around 60–70%). SAPs can be easily spotted by AFM (Paolini et al., 2016).

#### AI Ratio Actually Decreases Upon Dilution, but Suddenly Starts to Increase Again If I Continue to Dilute the Sample. Why?

Because you have diluted too much. High dilutions (namely, >1:500) are often counterproductive and lead to a “homeopathic” case similar to the intREF: irrelevant amount of EVs in solution and a modest decrease in AI ratio due to PBS. Therefore, it is advisable to stop diluting whenever the AI ratio falls below 20%.

#### How Do I Distinguish a Pure Sample Containing Vesicles From One With No Vesicles? The AI Ratio Decreases in Both Cases

Yes, but to a different extent. The intREF and dilutions help. Indeed, a preparation containing no EV will have an AI ratio similar to the intREF, and it will stay stable regardless of the dilution factor. We do note that the CONAN assay is not the proper technique to verify the presence/absence of EVs. This should be verified, for instance, by checking for proper biomarkers and imaging [as discussed in (Thery et al., 2018)].

## Advantages and Limitations

The CONAN assay has several advantages: (i) Unprecedented low cost and accessibility. To date, the molar concentration of EV preparations is typically performed by expensive (>70 k€) and dedicated lab instruments, for example, NTA, RPS, flow cytometry (Gardiner et al., 2016), which require tens to hundreds of microliters of EV preparation per measurement. The CONAN assay is accessible, requires very low sample volumes (1–2 μl/measurement), and has a colorimetric readout visible by the naked eye, which can be quickly quantified by a standard or a microplate UV-Vis reader (experimental time <1 h), and reagents are cost-effective and easy to prepare. (ii) The CONAN assay provides an index, that is, the AI ratio, to grade the EV formulation purity, which may exit the research lab and be implemented for standards and regulations. (iii) The titration phase of the CONAN assay only works with EV formulations that resulted pure. Therefore, purity assessment is the gatekeeper for the subsequent titration, providing a very convenient internal check. (iv) Robust dose-response linearity allows for a reliable batch-to-batch comparison and next use.

To the best of our knowledge, these limitations are all related to the titration phase: **(i)** A systematic error in the determination of the absolute value of the molar concentration is possible. This may primarily arise from inaccuracies in the liposome calibration line, for example, discrepancy between the nominal molar concentration of phospholipids and the effective number of liposomes, discrepancies between the liposome and EV size distributions, differences between the interaction of the liposomes and of the EVs with the AuNPs. Regarding this last point, the AFM analysis shown in Figure S3 suggests that the AuNP aggregation at the lipid membrane is fairly similar between liposomes and EVs. **(ii)** The EV mean size must be known to calculate the EV absolute molar concentration (**Note:** Indeed, size distribution is an indispensable parameter among the minimal information required for any EV sample; Thery et al., 2018). **(iii)** The CONAN assay (viz. the AuNPs) in the absence of SAP primarily targets the lipid membranes but may also interact to a certain extent with aggregated lipids (investigation in progress); therefore, the coexistence in the assayed solution of non-EV lipid nanoparticles, such as exomeres (Zhang et al., 2018) or lipoproteins (Yuana et al., 2014) may interfere with the assay's proper operation and response.

## Data Availability Statement

All datasets generated for this study are included in the article/Supplementary Material.

## Author Contributions

AZ, LP, SB, and PB contributed to conceptualization. MHMW, PN, and PB contributed to funding acquisition and contributed to supervision. PB contributed to project administration. MJCvH and AB contributed to resources. AZ contributed to visualization and contributed to writing the original draft. All authors contributed to investigation and methodology, writing, reviewing, and editing. Please refer to the CRediT taxonomy for the term explanation.

### Conflict of Interest

The authors declare that the research was conducted in the absence of any commercial or financial relationships that could be construed as a potential conflict of interest.
