# Corrigendum: Augmented COlorimetric NANoplasmonic (CONAN) Method for Grading Purity and Determine Concentration of EV Microliter Volume Solutions

**DOI:** 10.3389/fbioe.2021.674507

**Published:** 2021-04-13

**Authors:** Andrea Zendrini, Lucia Paolini, Sara Busatto, Annalisa Radeghieri, Miriam Romano, Marca H. M. Wauben, Martijn J. C. van Herwijnen, Peter Nejsum, Anne Borup, Andrea Ridolfi, Costanza Montis, Paolo Bergese

**Affiliations:** ^1^Department of Animal Science, Food and Nutrition-Università Cattolica del Sacro Cuore, Piacenza, Italy; ^2^Consorzio Interuniversitario Nazionale per la Scienza e la Tecnologia dei Materiali, Florence, Italy; ^3^Department of Molecular and Translational Medicine, University of Brescia, Brescia, Italy; ^4^Consorzio Sistemi a Grande Interfase, Department of Chemistry, University of Florence, Sesto Fiorentino, Italy; ^5^Department of Transplantation, Mayo Clinic, Jacksonville, FL, United States; ^6^Department of Biochemistry and Cell Biology, Faculty of Veterinary Medicine, Utrecht University, Utrecht, Netherlands; ^7^Department of Clinical Medicine, Faculty of Health, Aarhus University, Aarhus, Denmark; ^8^Istituto per lo Studio dei Materiali Nanostrutturati (CNR-ISMN), Bologna, Italy; ^9^Department of Chemistry, University of Florence, Sesto Fiorentino, Italy

**Keywords:** extracellular vesicles, synthetic vesicles, liposomes, purity, titration, particle number, nanoparticles, nanoplasmonics

In the original article, there was an error. The reported concentration of the trisodium citrate dihydrate solution, “3.5 mM,” used to synthesize gold nanoparticles (AuNPs), was incorrect. This error inevitably leads to failure in gold nanoparticle synthesis. The correct trisodium citrate dehydrate concentration should be “34.0 mM.”

A correction has been made to the **Methods** section, subsection **Preparatory Procedures**, paragraph, under the **AuNP Synthesis** method:

**AuNP Synthesis**

AuNPs are synthesized through classic Turkevich's citrate reduction method (Turkevich et al., 1951).

- Dissolve trisodium citrate · 2H_2_O in HPLC grade water to a final concentration of 34.0 mM (1.0% wt).
- Prepare 20 ml of 1 mM HAuCl_4_ in HPLC grade water.
- Boil the HAuCl_4_ solution under continuous stirring.
- Inject 2 ml of trisodium citrate · 2H_2_O 34.0 mM into the boiling solution. **Crucial step:** trisodium citrate injection speed influences AuNP size and therefore final concentration. A one-shot injection leads to smaller particles (usually around 12–15 nm); slow injection will result in NP aggregation and precipitation.
- Keep stirring and wait for the solution to change color from the original pale yellow to wine red.
- After 10 min, cool the solution in a water-ice bath for 5 min.
- Store the AuNPs at 4°C. **Crucial step:** 10–20 nM AuNP solutions are metastable and tend to form aggregates and precipitate with time. In our experience they keep their properties with respect to the assay up to 1 month of storage at the above described conditions. Anyhow it is strongly suggested to freshly (re)determine before any usage the key characteristics of the AuNP solution which are relevant for the assay as described in section Determination of AuNP Molar Concentration and Spectral Properties.

The authors apologize for this error and state that this does not change the scientific conclusions of the article in any way. The original article has been updated.
