# Supplementary material for: Augmented COlorimetric NANoplasmonic (CONAN) Method for Grading Purity and Determine Concentration of EV Microliter Volume Solutions
Source: Front Bioeng Biotechnol. 2020 Feb 12;7:452. doi: 10.3389/fbioe.2019.00452 (PMC7028770; doi:10.3389/fbioe.2019.00452)
Supplement: Supplementary file 1 [file Data_Sheet_1.pdf]

Supplementary material

"Augmented COlorimetric NANoplasmonic (CONAN) method for grading purity and determine concentration of EV microliter volume solutions"

Andrea Zendrini*[1,2], Lucia Paolini*[3,4], Sara Busatto[4,5], Annalisa Radeghieri[3,4], Miriam Romano[3,4], Marca H. M. Wauben[6], Martijn J. C. van Herwijnen[6], Peter Nejsum[7], Anne Borup[7], Andrea Ridolfi[4,8,9], Costanza Montis[4,9], Paolo Bergese[2,3,4]

[1] Department of Animal Science, Food and Nutrition - Università Cattolica del Sacro Cuore via Emilia Parmense 84 29122 Piacenza, Italy.
[2] Consorzio Interuniversitario Nazionale per la Scienza e la Tecnologia dei Materiali – via Giuseppe Giusti 9 50121 Firenze, Italy
[3] Department of Molecular and Translational Medicine - University of Brescia viale Europa 11, 25123 Brescia, Italy.
[4] Consorzio Sistemi a Grande Interfase, Department of Chemistry – University of Florence via della Lastruccia 3 50019 Sesto Fiorentino, Italy.
[5] Department of Transplantation, Mayo Clinic, Jacksonville, FL 32224, USA.
[6] Department of Biochemistry and Cell Biology, Faculty of Veterinary Medicine, Utrecht University, Utrecht, The Netherlands.
[7] Department of Clinical Medicine, Faculty of Health, Aarhus University, Palle-Juul Jensens Boulevard 99, 8220 Aarhus N, Denmark.
[8] Istituto per lo Studio dei Materiali Nanostrutturati (CNR-ISMN), via P. Gobetti 101, 40129 Bologna, Italy.
[9] Department of Chemistry - University of Florence via della Lastruccia 3, 50019 Sesto Fiorentino, Italy.

Use of a modified calibration curve for EV quantification

Since not all the spectrophotometers are able to collect absorbance spectra in the specified range (400-900 nm) we tested the possibility to calculate the AI of liposome standards using slightly modified versions of **eq. (1)**. Indeed, the use of the absorbance collected at 800 nm or 780 nm instead of the one collected at 850 nm causes minimal discrepancy in EV quantification. Such difference has been calculated to be < 1% for the samples used as working examples in the manuscript. Discrepancy sensibly grows whenever any absorbance value below Abs_{760nm} is used. **Figure S1** shows the comparison between the calibration curves plotted by replacing Abs_{850nm} with other absorbance values.

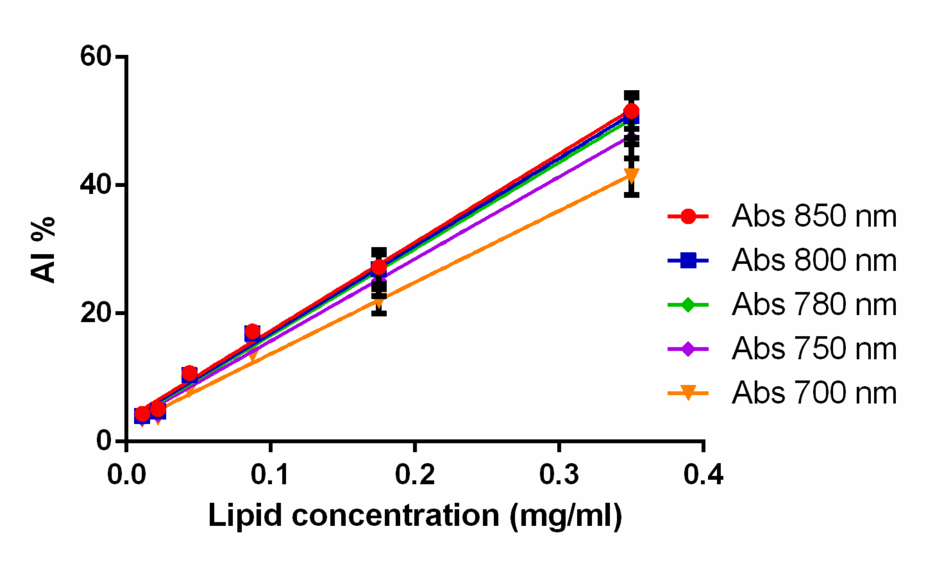

Figure S1. A comparison between the calibration curve obtained using the standard form of eq. (1) for *AI* calculation (red line) and the one obtained using its modified versions (blue, green, purple and orange lines).

CONAN assay single aggregated protein (SAP) limit of detection (LOD) definition

The experiment was performed to measure CONAN assay LOD for SAPs.

Soluble proteins block the interaction between lipid membranes and AuNPs, due to the formation of a protein corona on AuNP surface. Aggregation impairment is progressive and follows the concentration of SAPs. In this experiment, a fixed amount of liposomes (0.022 mg/ml of lipids, equal to the second point of calibration line showed in **Figure 2D** in the manuscript) was mixed with increasing concentration of BSA (0, 0.002, 0.005, 0.01, 0.02, 0.05, 0.1, 0.2, 0.5 and 1 µg/µl) and tested with CONAN assay, as described in **section** *EV purity check* in the main text. AI ratio was calculated using **eq. (2)**. Results of the experiments are shown in **Figure S2**.

AI ratio starts to increase sensibly at a concentration of SAPs comprised between 0.02 and 0.05 µg/µL (highlighted in yellow in **Figure S2**). Such range corresponds to an AI ratio of ~ 20%, which has been therefore selected as CONAN assay SAP LOD.

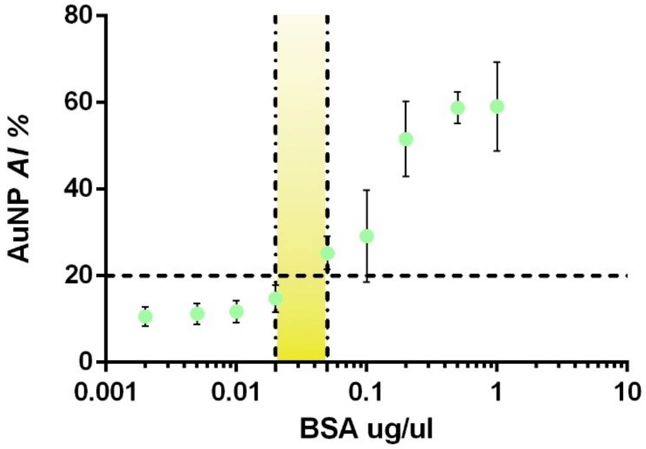

Figure S2. Determination of CONAN assay LOD for SAPs.

AFM imaging of pristine POPC liposomes and EVs before and after interaction with (citrate-capped) AuNPs

The experiment was performed to visualize the formation of AuNPs clusters on to lipid membrane of POPC liposomes and EVs. Liposomes and EVs were mixed with the same AuNPs (citrate-capped,~15 nm, 6 nM) used for CONAN assay and incubated at RT for 30 minutes. Five µL of liposomes or EVs were then deposited onto mica sheets and let to adsorb for 10 minutes at 4°C. The substrate was then inserted in the AFM fluid cell. AFM imaging was performed in ultrapure water at room temperature, using a Bruker Multimode 8 running in PeakForce mode equipped with Bruker SNL probes (nominal tip radius 2-10 nm, nominal spring constant 0.24 N/m). Image analysis was performed using Gwyddion 2.55 and custom Python scripts. Images **(A)** and **(C)** show a bare liposome and a AuNP-coated liposome, respectively. Images **(B)** and **(D)** show a bare EV and a AuNP-coated EV, respectively. The same particles are shown slightly magnified in the relative insets **(a), (b), (c),** and **(d)**. A single AuNP and relative Z-scale bar is shown in inset **(e)**.

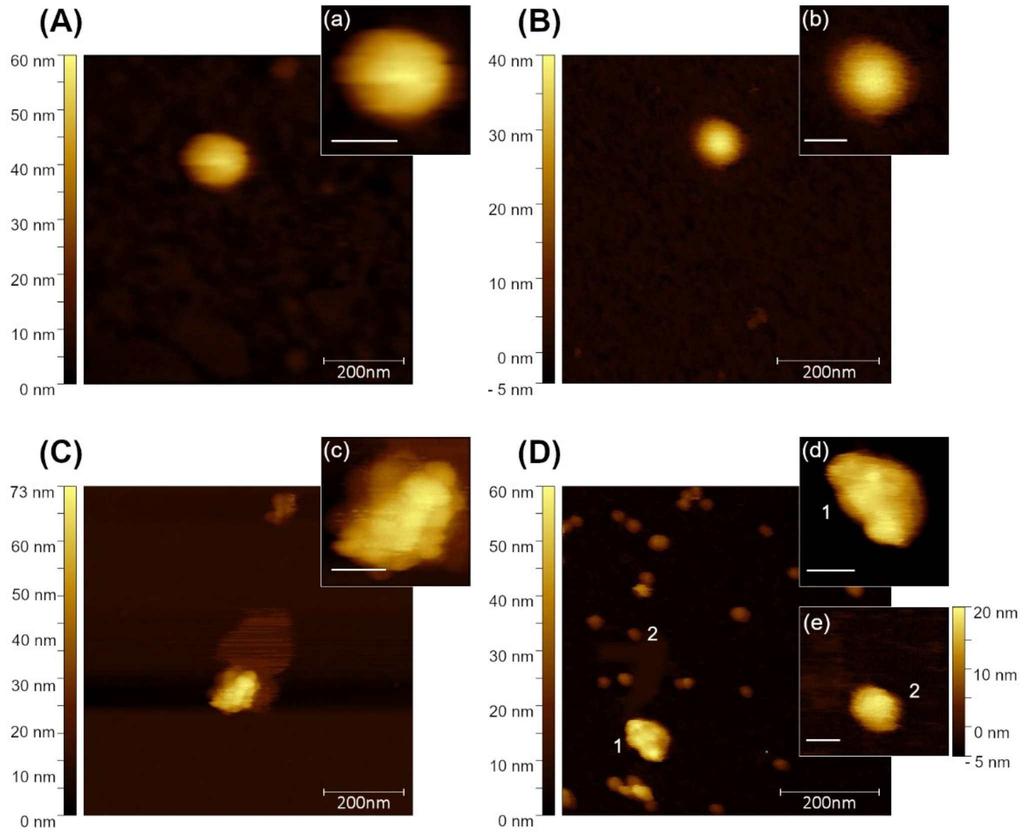

Figure S3. AFM analysis of POPC liposomes **(A)**, EVs **(B)**, AuNP-coated POPC liposomes **(C)** and AuNPs-coated EVs **(D)** (marked with number 1 in picture **(D)** and inset **(d)**). A zoom of the particles is shown in the relative insets. Inset **(e)** shows a single citrate-capped AuNP (marked with number 2 in picture **(D)**) imaged from picture **(D)**. Scan size 700 nm x 700 nm. XY scale-bar size 200 nm for images **(A)**, **(B)**, **(C)**, **(D)**, 50 nm for insets **(a)**, **(b)**, **(c)** and **(d)**, and 20 nm for inset **(e)**.

UV-Vis spectra of solvents used for liposome or EV resuspension

The UV-Vis absorption spectra of HPLC water, PBS, 0.9% NaCl solution and HPLC water-PBS and HPLC water-0.9% NaCl solution (in the proportion used in the CONAN assay) are reported in **Figure S4**. The differences in absorption spectra are negligible.

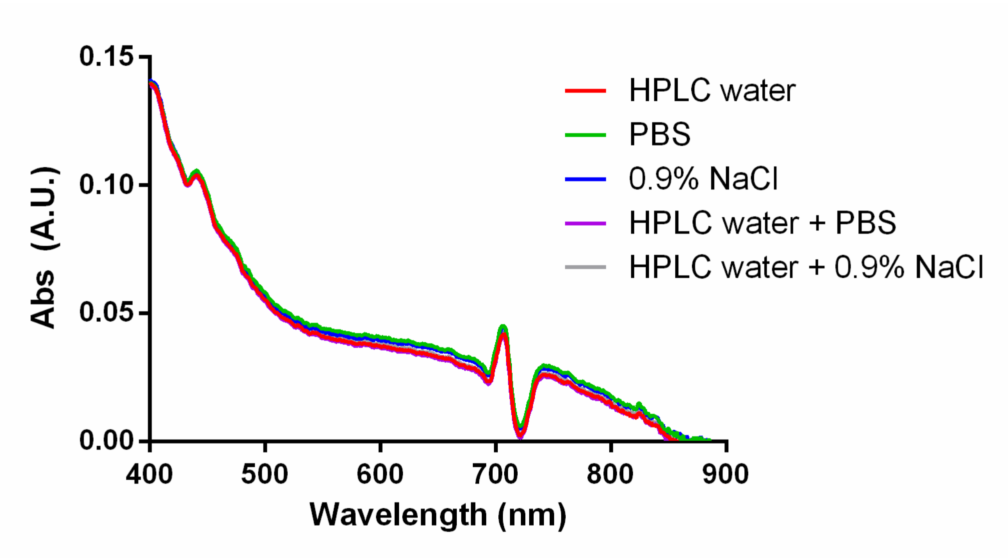

Figure S4. UV-Vis spectra of solvents used for liposome or EV resuspension: HPLC water (red line), PBS (green line), 0.9% NaCl solution (blue line), HPLC water-PBS (purple line) and HPLC water-0.9% NaCl solution (grey line). The differences in absorption spectra are negligible.

Datasets

Datasets generated for this study are shown in this section.

Lipid concentration mg/ml	AI ratio		
0.35	54.77881	49.37444	50.68038
0.175	29.62578	27.77955	24.3611
0.0875	17.75862	17.68823	16.18338
0.04375	10.80469	11.12988	10.26112
0.02187	5.270135	5.18036	4.827357
0.0109	4.390049	4.41227	4.269877

Table S1. Dataset of 3 different replicates used for the calibration line showed in **Figure 2D.**

	milk contaminated EVs 1:1	milk contaminated milk EVs 1:10	milk contaminated EVs 1:30	*A. suum* pureEVs 1:1	*A. suum* pure EVs 1:3	*A. suum* pure EVs 1:5
AI ratio replicate 1	64.31976	69.54499	69.74865	43.96085	10.97162	9.358689
AI ratio replicate 2	61.29842	62.37967	59.34306	32.79765	11.08384	9.772814
AI ratio replicate 3	69.68902	//	59.9722	45.05204	12.33872	10.7995

	normREF	intREF	milk pure EVs 1:1	milk pure EVs 1:10	milk pure EVs 1:30
AI ratio replicate 1	100	48.0285	71.16329	21.04964	18.60194
AI ratio replicate 2	//	52.921	76.97102	18.03322	7.996666
AI ratio replicate 3	//	47.8845	74.24379	22.33495	7.846751

Tables S2. Datasets of 3 different replicates used to plot CONAN assay graph shown in **Figure 3**.

BSA µg/µl	AI ratio								
0	15.06636	14.30291	13.82577	8.357368	7.470352	12.10804	14.97612	15.26518	14.66718
0.002	13.47745	12.5677	13.57003	9.547992	9.416581	11.55967	8.685877	8.33465	8.474903
0.005	12.3509	14.79192	14.91365	10.65521	11.34508	10.12346	9.068083	8.153257	9.617648
0.01	14.50453	15.45977	14.61107	9.286279	9.84427	12.44684	8.527717	10.32334	10.73204
0.02	16.08653	17.45072	17.11289	15.81552	16.44981	17.82657	10.66893	11.73777	9.504291
0.05	19.68529	21.52819	21.19415	27.23159	29.94349	27.20659	28.24414	26.78572	//
0.1	14.68987	16.03904	15.58114	32.24591	36.4959	39.31668	32.24591	36.4959	39.31668
0.2	52.39283	42.46384	52.66331	59.33555	59.34299	39.72411	59.33555	59.34299	39.72411
0.5	63.55705	55.19255	62.15158	59.54224	60.58446	54.01313	59.54224	60.58446	54.01313
1	41.35793	46.59412	69.43673	57.1846	55.40801	63.60318	67.2089	71.79967	59.21967

Table S3. Dataset of 9 replicates used to plot the graph shown in **Figure S2.**

Funding

This work was supported by the Center for Colloid and Surface Science (CSGI), Aarhus University and Utrecht University through the evFOUNDRY project, Horizon 2020-Future and emerging technologies (H2020-FETOPEN), ID: 801367.
